# Fertility control in ancient Rome

**DOI:** 10.1080/09612025.2020.1833491

**Published:** 2020-11-02

**Authors:** Rebecca Flemming

**Affiliations:** Faculty of Classics, University of Cambridge, Cambridge, England

## Abstract

This paper surveys and evaluates the range of methods recommended mostly to promote but also to prevent pregnancy in ancient Rome, and then discusses the practices of adult adoption and infant exposure in more detail in order to interrogate the notion of ‘fertility control’ from an ancient historical perspective. Is this formulation sufficiently flexible to encompass Roman procreative projects and the resources they were able to bring to bear on them? Were the methods deployed sufficiently effective to qualify as ‘control’, and was it ‘fertility’ that was being acted on through adoption and exposure? This essay answers these questions positively and argues that the Roman case has plenty to offer wider debates about the history of reproduction as it includes the desires to have and not to have children, to limit and increase offspring, to shape families in different ways.

One of the key shifts in the long history of human procreation is from societies in which the dominant fertility project was the production of healthy children to those in which the limitation of that production dominates. The causes of this transition continue to be debated, and the move is a complex one beyond questions of etiology.^1^ Globally the pace and patterning of change has been and remains uneven; both between and within the developed and developing world. Nor do the pressures and motivations around generation ever operate universally in any society, past or present. The reproduction of some groups is always enabled and encouraged more than others. Individual aims and circumstances vary: it is not as if there was no interest in contraceptives and abortion before the nineteenth century, nor is there an absence of those desperately seeking to have children now. Still, the broad move is clear.

This was not a shift from passivity to activity, from easy thoughtlessness to careful attention. The business of having healthy children had to be worked at consciously and strategically no less than the business of not having children. Agency in the fertility domain, particularly female agency, should not be restricted to action around contraception and abortion, but understood more holistically, as recent scholarship on medicine and childbearing in medieval Europe has emphasized.^2^ Studies of the historical demography of East Asia have increasingly found themselves occupying the space between Louis Henry’s foundational ‘natural’ and ‘controlled’ fertility regimes, a space characterized by deliberate family planning that none the less fails to meet the narrow requirements of the traditional parity-based model.^3^ Behavior was not bound simply to the number of children already born but also to their sex and survivorship, among other considerations.

Recognizing that in all cases families and individuals (as well as communities and states) had procreative aims toward which they consciously worked, in divergent historical circumstances and with different resources to call upon, entails a certain comparability across time and space. It suggests a long-term narrative in which the reproductive project itself, whether more expansive or restrictive, provides the unifying thread to be tracked and analyzed. ‘Fertility control’, as the subtitle of this volume indicates, might be the most useful formula to encompass these different possibilities under a single rubric, and allow a richer history of continuity and change to emerge: joining two words of sufficient flexibility and openness. It is not entirely unproblematic in its resonances, however, as noted in the concluding remarks of a recent attempt to take the long view of generation:

While helpful in linking the prevention and promotion of procreation, the term may be too modern for centuries before the twentieth. People have always aimed to achieve certain objectives for family continuity and population size, individual health and happiness, but their conceptual and practical tools have changed.^4^

‘Control’ is a modern reproductive term and has taken on some particular meanings in the late modern world more generally. It raises two sets of questions in relation to its broader applicability in this field. The first are practical, or empirical, the second more conceptual. There is an issue about whether ‘control’ sets the efficacy bar too high for the pre-modern world: whether, or to what extent, success in respect to or at least real purchase on the challenges and aims involved is required to use this language. Then there is the sense in which ‘control’ has now become an aim in itself rather than a means to an end, and so perhaps lacks the categorical stability necessary to do the requisite heuristic work. The two converge and overlap at various points too, interweaving with a further series of queries about the reach of ‘fertility’, which is where two practices this essay gives particular attention to—infant exposure and adult adoption—come in. Both actions were enabled by Roman law and ingrained in Roman society, and their effectiveness in limiting or ensuring family size is obvious. Their relationship to notions of fertility, on the other hand, seems strained by a mixture of temporal and somatic distance, which may also cross back over with ideas of control. Is this something which can be exercised after the event? Does it have to be exercised on physical fecundity, and focused around birth, or is that to construe a more complex phenomenon too narrowly?

These definitional issues are worth investigating further, as are the specificities of the historical practices themselves. The two perspectives need to be placed in dialogue. This article thus both elucidates aspects of the Roman world—the kinds of procreative strategies pursued within it and the resources available to support them—and contributes to the larger comparative project around ‘fertility control’. It proceeds in two parts, the first focused on questions of control, the second on what counts as fertility. The article will discuss the methods recommended to both prevent and promote pregnancy in ancient Rome, to be precise, in late Republican and early imperial Rome, over roughly the last century BC and the first two centuries AD, including an assessment of their effectiveness. Attention then turns to exposure and adoption, to the evidence for the ways they were thought about and practiced, as similar or different to other interventions around family size, before bringing the themes of fertility and control back together in conclusion.

## Fertility *control*

A little after AD 100, in Rome, the noted physician Soranus of Ephesus composed his *Gynecology*, the only such dedicated treatise to survive from the early Roman Empire.^5^ Like other ambitious doctors, Soranus had traveled from his provincial birthplace—the city of Ephesus in Asia—to the imperial capital, via the medical schools of Alexandria, and, though he spent most of his career in Italy, he continued to write in his native Greek. Still the dominant language of learned medicine, Greek works addressed not only other physicians but also a largely bilingual (or multilingual) Roman elite.^6^ The rich and powerful of Rome wanted to have healthy children and Soranus offered instructions about how to achieve that goal, advice which he constructed polemically, through opposition to and criticism of past medical authorities and present rivals. In particular, he positioned himself against the traditional Hippocratic view that female health depended on generation, arguing instead that women’s physical well-being was undermined by her ‘child-production’ (the literal translation of the Greek *teknopoia*).^7^ This production was necessary to ensure the ‘succession of beings’, the continuation of the species, but the task was more challenging than the majority of doctors admitted. It required greater attention to looking after the female body itself, to counteract the damage of childbearing.

Soranus’ pro-procreative program was thus complex but unremitting. It started with female anatomy and moved onto a systematic study of all the processes involved in generation, from menstruation to birth and the care of the newborn. Sex and marriage (synonymous for respectable Roman women) follow menstruation in this sequence, indeed Soranus insisted that girls pass menarche and become physically mature enough to sustain intercourse with a man and bear a child before being married.^8^ This advice was somewhat at odds with elite practice in the Roman empire, though his judgement that delaying matrimony too long was also dangerous would have been less contentious.^9^ He argued that questions about the fertility of any prospective bride—‘whether or not they are able to conceive (*sullambanein*) or have the right physical formation to give birth’—should accompany the customary inquiries ‘about the excellence of their lineage and the abundance of their wealth’ in assessing her suitability. His aristocratic audience, however, seem to have ignored this advice.^10^ All the evidence indicates that the Roman elite stuck to their traditional interests in birth, money and looks.^11^ Not that they were indifferent to fertility, but concrete matrimonial decisions were usually dictated by particular contingencies, by the conjunctural needs of family alliance, and there was a view that women’s childbearing prowess was something to be proved rather than guessed at. The only women who possess the virtue of ‘*fecunditas*’, in the *Annals* of the Roman historian Tacitus, for instance, have already born children.^12^

After the account of how to recognize the capacity for conception Soranus proceeded to provide detailed advice on the best time for procreative intercourse.^13^ What condition should a woman be in to optimize her chances of receiving and retaining the man’s seed, of being able to begin to nourish and mold what has been held in a proper fashion, as Soranus understood the process of conception (*sullēpsis* in Greek, literally ‘grasping’) itself.^14^ His answer followed the Hippocratic view that women are most likely to conceive as their periods are dwindling and stopping.^15^ The womb was at its most receptive at this juncture, warm and moist in good measure, turning from evacuative to accumulative mode but not yet congested and overburdened. For the rest, body and soul must be in the right condition, feeling good and appropriately inclined.

The question then is whether the basic sense in Soranus’ stress on a well-balanced body and a willing soul is outweighed by the mismatch between ancient and modern understandings of the relationship between fertility and menstrual periodicity. In modern medicine, the ‘fertile window’ refers to the six days during which heterosexual intercourse can result in pregnancy, those being the five days before and the day of ovulation itself. If ovulation occurs exactly halfway through a standard twenty-eight-day cycle, then the window would be between days ten and seventeen (counting commences on the first day of bleeding), which clearly does not align with Soranus’ best time. Recent work has emphasized variability in respect to both menstrual and ovulatory cycles, however, and there has been something of a forward drift in fertility, thus rendering Soranus’ advice less problematic.^16^ More importantly, it was never intended to be exclusive. These specific instructions were located within a wider frame of assumed marital intercourse.

With conception complete, or at least well underway, guidance on care for the pregnant woman followed. It had three stages—the first aimed at guarding the deposited seed, the second at alleviating the ensuing symptoms, such as those associated with *kissa* (characterized by cravings, nausea, and general digestive disarray), and the last, as lying-in approaches, aimed at perfecting the embryo and preparing for the demands of birth.^17^ Every aspect of the woman’s life was to be regulated, from what she ate and drank to the frequency of her baths; her emotional and physical range was to be restricted, her thoughts and actions modulated. The main message was to take things easy—not too easy, gentle exercise was mandatory—and eat well, while avoiding shocks and traumas, excess and anything harmful.

Given the agenda so far, it is perhaps surprising that the first book of the *Gynecology* ends with a chapter on contraception and abortion. However, as mentioned, Soranus also explicitly brought out the detrimental effects on women of all these processes: menstruation, sexual intercourse, conception and pregnancy. Childbearing uses up resources, saps vigor, and causes premature aging: ‘just like with the earth, which becomes so exhausted from continuous fruit-production that it is not able to carry fruit every year’.^18^ Soranus thus opened up conceptual space in which talk of family limitation could occur, within the pro-procreative program. There are the woman’s interests to be balanced against the need for family continuity and while the latter has priority, some allowance can be made for the former without compromising the overall project.

The chapter opens by distinguishing contraceptives and abortives, differentiating between those items and actions which prevent conception (*sullēpsis*) and those which destroy what has been conceived.^19^ These latter were called ‘destroyers’ (*phthoria*) with the former termed ‘non-birthers’ (*atokia*). Destruction of what is carried has been controversial, Soranus reported, with some physicians opposed to any such interventions while others argued for a discriminating approach. The opposition called Hippocrates as a witness, who said ‘I will give no woman an abortive’, and asserted that the medical art must guard and preserve what has been generated by nature.^20^ The proponents of judgement explained that they would not assist a woman who wished to destroy what she had conceived on account of adultery or vanity but rather to prevent dangers in birth caused by the womb being too small, or by calluses or fissures of its mouth, or any similar difficulties. They said the same about contraceptives, and Soranus concurred. He placed himself firmly in the camp of those willing to prescribe both *atokia* and *phthoria* in the appropriate circumstances, preferring the first, since prevention was safer than destruction.

Soranus’ contraceptive prescriptions can be roughly divided into three. The first clustered around the act of intercourse itself.^21^ The ‘best time’ for procreative sex should be avoided, the woman should move away as the man is about to ejaculate, or get up immediately afterwards and encourage the seed to leave her body by squatting, sneezing or other actions. The second involved applications to the mouth of the womb prior to intercourse.^22^ Substances such as old olive oil or a moist cerate of myrtle oil and white lead can be externally applied to assist in ‘non-conception’ (*asullēpsia*) or pessaries composed of items to close up the womb or heat and irritate it, preventing the entry or retention of the seed respectively, can be inserted and then removed before sex. Soranus provided several recipes, with pomegranate rind, oak gall, and various minerals the most favored ingredients. Last were what might be termed oral contraceptives.^23^ Plant materials—seeds (especially rue seeds) and balms—are ingested monthly with liquids. These things destroy as well as prevent, Soranus concluded, *sullēpsis* was, after all, rather a complex process with a vague finishing point, and they are damaging to the body.^24^ Soranus’ discussion of abortives followed a similar pattern.^25^ For the thirty days after conception do the opposite of what he advised to guard the deposited seed. The woman should jump around, carry heavy loads, eat the wrong foods, attempt purges and take long baths. The next stage of intervention involved more medicinally potent baths—with linseed, mallow, wormwood and rue plants in them, for example—together with the application of similarly composed poultices and enemas. Last, women could be extensively bled, or abortive pessaries resorted to. Avoid anything too powerful, however, and any kind of physical removal with sharp objects, for wounding the surrounding area is dangerous.

Several of the ingredients listed here have been identified as having fertility suppressing effects in a range of ethnobotanical and laboratory studies. John Riddle was the first to survey this evidence in relation to ancient medical writings and to argue very strongly that knowledge of effective contraceptives and abortifacients was widespread in antiquity.^26^ His work has been subject to sustained criticism ever since: its orientation, presuppositions, methodology and conclusions have all been called into question.^27^ Discovering what modern species might be designated by ancient plant names is far from straightforward, for example, while experiments showing that feeding rats large amounts of pomegranate rind decreased their fertility by almost 30 percent may reveal nothing about its impact on human women when applied in pessary form. Still, the possibility of efficacy must be allowed for, as burgeoning global research into traditional herbal remedies—including those aimed at generation—indicate.^28^ This is, of course, efficacy broadly construed, as meaningful effect rather than the guaranteed success demanded by modern biomedicine, but it seems likely that some of Soranus’ prescriptions would have diminished fertility to some degree.^29^

More importantly, Soranus explicitly located his discussion of contraceptives and abortives within marriage. Traditionally, though recipes and substances might simply be labeled, ‘*atokia*’, or even ‘*phthoria*’, in pharmacological contexts or works on medical materials, actual engagement with the business of prevention or destruction occurred in association with prostitution. Soranus himself referred to the case of the enslaved ‘entertainer’, made to expel the ‘seed’ (*gonē*) she had retained following intercourse by the Hippocratic physician who authored *On the Nature of the Child*.^30^ The philosophical poet Lucretius, writing his Latin epic *On the Nature of Things* in the last decades of the Roman Republic, had asserted that women themselves can ‘prevent or resist’ conception, by pulling away and becoming limp as a man climaxes.^31^ This technique belongs, however, to ‘whores’ (the Latin is the more pejorative, *scorta*), who wish to minimize their chances of becoming pregnant and maximize their client’s pleasure. It is not the business of ‘our wives’. They were there for the production of legitimate children, while prostitutes’ role was the production of legitimate male sexual pleasure, a legitimacy predicated on the separation of the transaction from procreation: that it was not generative in itself and did not compromise other men’s family strategies.^32^ Soranus, however, wanted to make methods of non-conception, and even abortion, available to wives, under the pro-natalist banner.

The second book of the *Gynecology* covers the business of normal birth and the ensuing care of both mother and baby. For the purposes of this discussion there are two important points in the detailed descriptions and instructions. First and foremost is the section on how the midwife (*maia* in Greek) was to recognize whether the infant she had just delivered was fit for rearing or not.^33^ The main positive indicators were that the mother had enjoyed good health during pregnancy, birth had occurred at the proper time, the newborn had cried vigorously when placed on the ground, and was well-formed in all its parts, had good movement and sensitivity all over their body. If these criteria were not met then the midwife was to adjudge the infant unfit for rearing, too weak to survive, though more qualitative considerations may have entered the frame around formation and function too. In any case, the midwife was undertaking an essentially physical assessment that would contribute to but not necessarily determine the father’s decision to rear or expose—that is to put the new-born out to die or be picked up and raised by someone else. This will be examined more fully below, for now it is sufficient to note the way Soranus’ medical narrative engages with this critical social moment. The other issue of interest is the nutrition of the newborn. On a pragmatic level, Soranus favored wet-nursing over maternal breastfeeding and offered advice accordingly.^34^ He aligned himself with the dominant elite practice of the early Empire, and against arguments by some philosophers and traditional moralists that women should nurse their own infants.^35^ The employment of wet-nurses has, of course, practical implications for the possibility of birth-spacing in these aristocratic families, though outside those circles mothers were generally assumed to breast-feed their own babies.

The latter half of the *Gynecology* deals with the diseases of women, in which the dangers and damaging impact of pregnancy and parturition loom large. It is not just that discussion of difficult birth—*dustokia*—dominates the fourth and final book, but that the experience of such travails, along with miscarriages, are the most frequent causes of many of the pathologies described, particularly of the womb.^36^ There is even a condition termed the ‘exhausted’ (or perhaps ‘debilitated’) womb, caused by frequent pregnancies, stretching, and, especially, large embryos.^37^ It renders the uterus almost entirely unfit for procreative duties.

The sections on several of these uterine ailments are not preserved in their original Greek, but, apart from their headings, survive only in the later ‘Latinizations’ of the *Gynecology* by the fifth-century AD North African physician Caelius Aurelianus and his less firmly located successor Muscio (or Mustio).^38^ Similarly, the contents of the final chapter in book three of Soranus’ composition, listed as ‘On non-generation (*agonia*) and non-conception (*asullēpsia*)’ are transmitted only in Latin.^39^ Despite variations in vocabulary and construction, the message is the same in both versions. ‘*Sterilitas*’, the Latin for infertility or barrenness, accompanies or arises from a range of affections. It may be that ‘the seed is not received, or having been received it is not retained or having been nourished it is not perfected’.^40^ Though all of these failures occur in the female body, the cause may lie with either party, and may be a problem of the whole body or of the particular parts. The man can be too ill or weak to produce sound seed or have a malformed penis which prevents him from ejaculating in the right area, while the woman may be too thin or feeble or too fat or dense, for example, or have a misaligned, obstructed or injured uterine opening. All these complaints can be treated, mostly dietetically if addressing the overall somatic condition, and through pharmacological applications or surgery if the problem is more localized and specific.

Little detail about these therapies is offered, adding to the difficulty in assessing efficacy. The emphasis on general health seems more promising than many of the more specific interventions, though the disruptive conditions which can be fixed that way are limited. It is, however, worth bearing a couple of modern statistics in mind here. One, is that, in contrast to the 91 percent effectiveness of the contraceptive pill in Britain today, the current success rate for a cycle of IVF is only 29 percent in women under thirty-five and it drops pretty precipitously after that.^41^ The second, interrelated point is about the causal complexity and uncertainty that surrounds infertility. Modern studies implicate biological, behavioral, psychological and sociocultural factors, in a range of combinations, differently distributed in a partnership, and in 15–25 percent of cases, no physiological dysfunction can be identified at all.^42^ So, it may be that simply addressing the problem, doing something which was thought to help, would have had beneficial results.

There were non-medical courses of action available to those struggling to have children in the Roman Empire. One such avenue did find physiological support (though missing from the Latinizations of Soranus) in the standard recognition in ancient medical and philosophical discussions that infertility could be relational. Generative failure could be caused by some sort of incongruity or incompatibility between the couple having intercourse. The flaw in the partnership was variously construed—as a mismatch of bodies or sexual pace, of constitutions or seeds—but the suggested remedy stayed the same.^43^ Changing partners might bring better results. The formulations were mostly vague, but Lucretius clearly recommended divorce and remarriage, repeatedly if necessary, in contexts where no progeny had been forthcoming.^44^ Indeed, he referred to both men and woman in previously barren unions who had subsequently found spouses with whom they had sweet and dutiful children.

By the time Lucretius wrote his didactic epic in the first century BC, divorce and remarriage were legally (if not practically) straightforward for both parties at Rome, especially if there were no surviving offspring.^45^ There was some debate about the propriety of divorcing a loyal and virtuous wife solely on the grounds of procreative failure, at least if she did not agree, but it was perfectly possible to do so and the absence of children made the logistics of separation easier.^46^ This was, moreover, simply a variation on a key theme in Roman matrimony—the main reason for divorce in the late Republic and early empire was to remarry, for political, economic, or generative purposes, maybe a combination of them all.

If Soranus’ apparent omission of the relational aspects of infertility is puzzling, his failure to mention the non-medical practitioners who provided procreative advice and assistance in the Roman world is more understandable. A range of texts from the imperial period demonstrate that dream interpreters, astrologers and fortune-tellers were often consulted about the production of children, pregnancy, birth and the prospects of the new-born.^47^ Stories of such encounters from the client’s perspective are missing from the record, but there are plenty of literary allusions to the general but problematic rise of private divination in the early empire.^48^ Soranus’ silence on those who might be considered his competitors for the attention and largesse of the elite is hardly surprising.

Much more could be said on these options, and on the possibility of direct appeal to the gods for help in having healthy children, but the point here is simply to return to the pro-procreative shape of Roman society, with which this section opened, having sketched out some of its particular lineaments and complexities. The range of resources—legal, medical, cultural and religious—available to those pursuing their particular family strategies within this frame has been part of this picture. These resources, moreover, certainly meet the requirement of real purchase and impact on the generative projects involved, and while not all were accessible to those below the elite, many were, at least in some form. Maximum effectiveness still resides in infant exposure and adult adoption, however, so it is to these phenomena that the discussion now turns.

## *Fertility* control

As Soranus assumed, in the Roman world birth was followed by a decision about whether to rear the newborn. A positive judgement meant that the processes of welcoming the child into the family and community would begin, while a negative one entailed the opposite, the separation of the child from their natal family through exposure, their being put out (*expositio* in Latin, *ekthesis* in Greek) either to die or be picked up and raised by someone else.^49^ It should be stressed that both possibilities were real, though the main reason for third party rescue was to bring up the infant as a slave. Exposure is, therefore, to be distinguished from infanticide: it was about separation, or rejection, not about the fate of the child.^50^ It was a means of regulating family size and family composition.

Soranus described a physical assessment of suitability to rear, one that was entirely gender neutral, but other ancient sources and modern scholarship raise the possibility of selectivity by sex in these post-parturition judgements, a selectivity that favored boys over girls. As Judith Evans Grubbs has explained, however, ‘the case for widespread exposure of females has been hugely overblown’, and, indeed, archaeology also demonstrates that at least some infants who would have failed Soranus’ fitness test were brought up.^51^ Issues of sex and disability surely played a role in Roman decision making about raising children, but in complex and relative rather than absolute ways.

Control can be exercised over quantity and quality, of course, and the efficacy of *expositio* is obvious in respect to both. It allowed the number of children in a family to be limited, and decisions to be made about a balance of girls and boys (or not). Indeed, until the development of reliable fetal sex discernment tests in the twentieth century, exposure and infanticide were the only means of sex selection in relation to offspring. The question is whether that control should be understood to have been exercised over fertility as well as family. The demographic orthodoxy would seem to be not, though some have assumed and Fabian Drixler has argued otherwise, at least for infanticide in early modern Japan.^52^ Scholars have also raised wider problems with solely parity-based definitions of fertility, so further consideration is required. Here the focus will be on whether the Roman sources themselves included *expositio* with other forms of family limitation or considered it as a distinct practice. Where did it fit in the overall demographic system of the Roman world?

Soranus’ approach was essentially inclusive, covering contraception, abortion, and exposure, as well as infertility treatments, in a single treatise. His discussion around raising was carefully circumscribed, however, limited by the role of the *maia* as reporter of the newborn’s physical condition to those in the family who would make the actual decision: most critically, the father, in whose power (*patria potestas*) any child raised would most likely be.^53^ Other factors would have been taken into consideration at this point, outside the purview of medicine or midwifery, with familial economics (broadly construed) most frequently mentioned in the sources. This came in two forms, one relating to the poor, the other to those with sufficient resources not to have to worry about an extra mouth to feed as such, but who were more concerned about the workings of a partible inheritance system in a world in which inherited wealth was key. Roman law made all legitimate offspring, female and male, automatic heirs (*sui heredes*) who had to be left a fair share of the estate unless explicitly disinherited.^54^ Bringing up more children could therefore be understood as diminishing the financial prospects of those already integrated into the family, though this was not the only way of thinking, and there were risks involved in deciding not to rear those born later even if the older offspring had already passed the most dangerous years of life.

A couple of decades before Soranus was writing, the Stoic moralist Musonius Rufus argued strongly in support of the thesis that all children born should be raised, which was more or less the position of the Stoa.^55^ Musonius reserved his greatest ire for those who ‘do not even have poverty as an excuse’ for exposing their infants, but who decide not to rear their later-born offspring ‘so that those earlier-born may inherit greater wealth’. As a rich Roman, as well as a philosopher, he would have been familiar with such practices, and the theme is repeated elsewhere. The most detailed story appears in a Greek novel of the second century AD, *Daphnis and Chloe*, both of whom are *expositi*, raised as slaves, providentially recovered by their parents so that they can end up happily married. Daphnis’ father, Dionysophanes, explained that, having married young, he was already lucky enough to have two sons and a daughter when the fourth child, another boy, was born.^56^ He thought his family ‘was big enough’, and so had the infant put out, a decision he later regretted, as his eldest son and his daughter subsequently died on a single day from the same illness. Even in his joy at finding his abandoned child, glad that he and his wife would have more support in their old age, Dionysophanes sought to reassure his other surviving son that his estate was substantial enough to make both of his children rich men. Though the first-born, Chloe had been exposed so that her father could continue to make the public expenditure required to maintain his civic status, again a matter of regret, since the expected future offspring failed to materialize.^57^

To return to Musonius Rufus, however, it should be stressed that his argument was essentially a civic one. Having lots of children was an obligation citizens owed to the state and the gods, though the benefits accrued to both the community and the family concerned, far outweighing the pragmatic excuses for limiting offspring that he dealt with. Failure to raise children who have been born was the dominant means to that impious and detrimental limitation, and so the primary point of attack, but Musonius also praised a variety of measures against abortion and contraception, public rewards for the parents of multiple progeny and penalties for the childless. The focus was thus fertility; he was opposed to all deliberate attempts to restrict the number of children produced and kept in a citizen marriage, favoring both discursive and practical encouragement to large-scale childbearing. He clearly considered exposure to be the main threat to his maximizing drive but as part of a wider set of practices with the same aims and outcomes.

*Expositio* was not just about family limitation, at least not directly. The end of marriage, through death or divorce, could have resulted in the exposure of any progeny born in the aftermath. Both pragmatic and emotional reasons seem to have been in play here, including matters of inheritance, once again. Some reported decisions appear strategic, such as the ‘clean-break’ agreement between a pregnant widow and her former mother-in-law recorded on a papyrus dated to 8 BC: the first acknowledged the return of her dowry, surrendered any further claims on her husband’s estate, and was then permitted to put out the child and remarry.^58^ Others look more impetuous, such as the second century AD case of a divorced wife who did not even tell her ex-husband (who had quickly remarried) about her pregnancy, electing to expose the baby instead.^59^ Then there is the question of ‘fatherless’ children, those born to a woman not in a Roman marriage (*iustum matrimonium*), so who were not born in *patria potestas* with all that entailed. Most of these would still have been engendered in a relationship which provided them with recognition and support, mostly marriages between persons (such as a Roman citizen and a free non-citizen) who could not contract a full *iustum matrimonium* under Roman law. These were not babies born to a ‘single’ woman, one who society deemed should not be having children or was having them by the wrong man, for example in adultery.^60^ So, though those latter women would likely have exposed their offspring, the numbers involved were probably small.

From whatever sources, however, sufficient numbers of newborns were exposed and then picked up by others that the raising of foundlings, a kind of ‘fostering’, became a regular and to some extent regulated occurrence in the Roman world.^61^ In contrast to later periods, there were no locations designated to receive abandoned infants, and no state or charitable institutions involved in their reception.^62^ It seems, rather, that certain local places became informally known as spots where newborns would be put out and could be taken up, by anyone who wanted to. As already stated, the main destination for *expositi* was slavery: exposed infants were picked up to be raised as slaves by individuals and in a more organized, business-like manner. The other possibility consistently mentioned in the sources is that *expositi* might be smuggled into reasonably wealthy, even positively elite households lacking offspring and presented as the product of their marriages by wives unable or unwilling to bear children for themselves. Another Stoic philosopher, and older contemporary of Soranus, Dio Chrysostom, referred to the former situation, not unsympathetically, for example. ^63^ Around the same time, the satirist Juvenal viciously attacked wealthy women allegedly reluctant to bear the burdens of pregnancy or the pains and perils of giving birth, thus fostering the obnoxious traffic in suppositious children, obnoxious because of the men fooled and the aristocratic lines thus sullied.^64^ Legislation and juristic discussions condemned the practice—there was no time-limit on fraud accusations concerning the introduction of such children, for instance—but they also recognized that husbands might collude in such undertakings as well as being their primary victims.^65^

The other significant legal interaction with exposure on the acquisition side related to the possibility that the foundling might be reclaimed, for freedom, for their natal family, or both. This may sound rather counter-intuitive, given the intimate association of *expositio* and slavery in the Roman Empire but, under classical Roman law, exposure did not affect the birth status of the infant.^66^ It remained free if born to a freewoman, and remained in *patria potestas* if that woman was in a Roman marriage. Now slave dealers could have moved *expositi* around, to ensure their ignorance of their origins and distance from any who did know and might have been willing to act on their behalf, adding further obstacles to a system already stacked in their own favor. Surely some did, but an alternative approach available to ordinary individuals and organized slavers also developed at least in some areas of the Roman Empire. It allowed these redemptions as long as the person who had raised the foundling was compensated for what they had spent on maintenance. Successive imperial rulers were asked to decide on this conflict between legal principle—of absolute continuity of status—and more pragmatic local custom, and while some had permitted this kind of purchase of freedom to be enforced in parts of Greece, the emperor Trajan preferred the principle. In AD 111 he responded to a letter from Pliny the Younger, governor of Bithynia-Pontus, which cast the dispute about the status and maintenance of ‘those called *threptoi*’ (foster-children) as one which affected ‘the whole province’, stressing the inviolability of free birth.^67^ If that status were proven, end of story, they should not have to ‘buy back their freedom’.

The scenario described by Pliny was not one in which the freeborn status of the *threptoi* was disputed; the issue was simply the payment of compensation. The situation was, therefore, characterized by knowledge of what had happened, not ignorance or concealment, whether that knowledge belonged to the parents, the rescuer, some third party, or was shared by them all. As Evans Grubbs argues, this openness changes how the practice, or at least some versions of it, should be understood.^68^ It suggests that some *expositi* did return to their original homes, even if not in the idyllic way imagined in *Daphnis and Chloe*. In fact, that may have been the plan all along. This chimes with the idea that among the (married) poor exposure was mostly a response to a specific crisis—such as crop failure, for whatever reason, or internal family disaster—rather than to poverty as such. For ordinary Romans, children were, despite the initial outlays, economically valuable as well as socially and culturally invaluable, as Saskia Hin has demonstrated.^69^ If desperate circumstances compelled them to put out a newborn it may well have been in the hope (though not the expectation) of future recovery, when things had improved, thus locating *expositio* among the adaptive strategies developed to spread the burden of childbearing and improve procreative outcomes as well as among the methods of family limitation. For the wealthy and ‘single’ women, the contexts were different.

Through discussing the redistributive, circulatory, aspects of exposure, the overlap with adoption has become apparent. Attention switches from the loss to the gain column of the family ledger and the transaction becomes more formal, stable and secure but the basic pattern is shared. In Roman adoption, a man who lacked a direct heir could acquire one, more or less fully formed, from another lineage to inherit his family name and cult as well as property.^70^ More complex heirship strategies and specific political aims could also be pursued by this means, but the rich surviving juristic discourse makes it clear that supporting family continuity was central to the institution, and adoptive households should roughly replicate natural ones.^71^ The model adopter was over sixty or otherwise known to be unable to procreate, had tried to have and maintain his own children, without lasting success. He should adopt an adult male at least eighteen years his junior, of similar social status if not actually part of the same kin group.^72^ The adoptee should also come from a family which could bear his transfer elsewhere, indeed his move would ideally benefit both parties. So, for example, the Terentius who features in one of the exempla collected by Valerius Maximus in the early first century AD had raised eight sons to young adulthood and gave one in adoption, intending that they should all be enriched by his inheritance.^73^ Even more exemplary was the case of Lucius Aemilius Paulus, scion of a noble house and victor over the Macedonian king Perseus at the decisive battle of Pydna in 167 BC, whose story was often retold. Earlier he had given up the older two of his four sons for adoption, ‘from abundance’ said Valerius Maximus, and to provide heirs for childless branches of two other illustrious families of Republican Rome—the Fabii and the Scipiones.^74^ While the adopted sons flourished, the younger boys died, a few days on either side of the triumph he celebrated for the victory at Pydna.

The narrative is a poignant one, often embellished with a speech in which Paulus expressed the view that his personal catastrophe was a counterbalance to the excessive good fortune he enjoyed in the service of the state. The adoptions did their job, however. Fabius and Scipio were able to transmit their ‘names, rites and households’ to outstanding heirs. They achieved their objectives in respect to their families. Their adopted sons also maintained links with their natal father, links strengthened by the particular circumstances as well as being part of the underlying structure, one of a number of signs that adoption was not the same as birth, that these were distinct ways of constituting households. Adopted children were legally in the same relationship to their *paterfamilias* as children who had been born to him in a legitimate marriage, but they had been raised by someone else. That raising, the emotional and material resources invested in it, its formative effects, the physical and moral resemblance between parents and offspring it forged, left its mark and was neither wiped out nor replaced by the formal transfer to a new family. The ideal, moreover, was continuity, of birth, rearing and inheritance: natural children were the preferred option, adoption was the second choice, effective but not the same in a social or personal sense.^75^

Less formal practices of fostering, of raising the offspring of others, might produce closer emotional ties, but without the same legal results: foster-children could not be heirs in the same way that adopted sons were.^76^ Adoption was a transaction involving Roman citizens, which required the agreement of both parties—the adopter and the father of the adoptee (the consent of the adoptee was relevant only if his father was dead). In the absence of any of those conditions raising a child without legally integrating them into the family was the only option, and there were a range of reasons why that course might be followed anyway, particularly below the elite. This also meant that any offspring born to a master by his slave women, since they followed the status of the mother, could not be adopted, and while it would have been theoretically possible to adopt children produced outside marriage, if the mother were a citizen (freed or otherwise), there is no evidence that this happened. The position of the adoptee, at least in elite circles, would have been socially untenable, and his inheritance would undoubtedly have been challenged in the courts, with some chance of success.

The Roman focus on adopting adults rather than young children puts a greater distance between adoptive and ‘natural’ families than in most modern societies and makes it more of a stretch to include the practice under the banner of ‘fertility’. The stretch may still be worth it, however, for, past or present, adoption is clearly part of a joined-up system that has fertility as its substance. The suggestion that the increased success of Assisted Reproductive Technologies is responsible for recent falls in the number of adoptions in the UK, for example, demonstrates the basic linkage.^77^ Ancient Roman decisions about whether to try to have or raise a child or not were surely informed by the presence of the institution of adult adoption. It cut both ways in these considerations. The main role may have been as insurance against future losses, thus underwriting a lower fertility regime than might otherwise be expected, as in various East Asian contexts. But the practice also offered support for extra offspring, having sons who could be beneficially given away to other families. The entanglements between adoption and fertility were many and varied.

## Conclusions

This essay has outlined the procreative projects pursued by the population of the Roman world in relation to the resources available to support and facilitate them. The aim has been to enable a fuller assessment of questions of control over those matters, as part of a longer history of fertility control. To summarize, everybody was in the business of family continuity, of having children to pass their name, status, cult and whatever property they might have owned on to, of forging links to posterity. The slaves who have, like others outside the group of elite Roman citizens, flitted in and out of this analysis, pursued a particular version of this project: wanting to have free children, to establish and then enact the possibility of family continuity after a period of generalized, definitional lack of control, including over their fertility.^78^ This and other more specific generative aims and contingencies operative in the Roman world deserve more attention in their own right but for the moment the focus will be on what was more or less shared across the board.

These broad family objectives were widely achieved, though keeping the offspring produced healthy was an ongoing challenge and some losses were almost inevitable for everyone. If production itself was a problem then divorce and remarriage, appeals for divine assistance and medical treatments were all possible courses of action, which could be selected, combined and repeated according to inclination and means. None of these options offered any guarantees but all improved the chances of success to some extent in a culture that studiously avoided blame for reproductive failure. Adult adoption did provide a guarantee, though of a slightly different form of family continuity, more openly instrumental and less ideal. Still, overall, it is hard not to see a comparable level of control operating in the Roman context as today in respect to the positive side of the equation, to having children. While adult adoption certainly stretches what might be considered a more traditional definition of fertility, so does modern child adoption, and various forms of surrogacy, all of which are clearly part of a single system through which progeny are currently generated and distributed. Not to consider these different actions around fertility together would seem to be a serious mistake, for both present and past.

Turning to more specific projects about family size and composition, it is important to distinguish between the elite and the rest. For the vast majority, while there would have been definite advantages to birth spacing, achieved through abstinence and breastfeeding, advantages for the prospects of the children as well as for overall family well-being, absolute limits were not an issue. Parents seem generally to have wanted both sons and daughters, a son first and foremost to ensure the continuity of the paternal line but also daughters, who made a range of important contributions to the family enterprise. It is likely that, if there were Roman data comparable to that from various historical periods in China, a similar pattern would emerge of women continuing to bear children for longer if they had either only daughters or sons among their preceding offspring.^79^ Sex-selective exposure might have been deployed in such circumstances, but decisions not to raise children were largely in response to crisis, albeit in a precarious world, where food shortages and famine were not infrequent, and with some wishful hopes of retrieving those given up when fortunes improved.

Among the elite, the pressures were greater, and strategizing around inheritance more developed.^80^ Birth spacing was neither sufficient nor so easily organized, given the reliance on wet-nurses; a pattern of rapid generation, of some sons and daughters, and then stopping, with the possibility of re-starting after either child mortality or a new marriage was more suited to family needs. This was harder to arrange, and though Soranus attempted to facilitate it through making contraception and abortion available to respectable married women and not just prostitutes, to protect those women from the most damaging effects of repeated childbearing, his recommendations would have been of limited efficacy, in respect to either pregnancy or well-being. Control would have come from abstinence or exposure, ultimately relying on the latter, without any benefits to female health. This was, as Musonius indicated, the dominant means of family limitation but one that operated within a wider suite of actions with the same aims, all of which he opposed while promoting moves encouraging childbearing. It is then not just measures to either support or restrict procreation which went together. Both were joined under the banner of fertility and its control.

## Notes

1 See e.g. A.J. Coale and S.C. Watkins (eds.), *The Decline of Fertility in Europe* (Princeton: Princeton University Press, 1986); John C. Caldwell, *Demographic Transition Theory* (Dordrecht: Springer, 2006); John Bongaarts and John Casterline (eds.), *Fertility Transition in Sub-Sahara Africa*, Population and Development Review 43: Supplement (New York: Population Council, 2017).

2 Monica H. Green, *Making Women’s Medicine Masculine: The Rise of Male Authority in Pre-modern Gynaecology* (Oxford: Oxford University Press, 2008); Katharine Park, ‘Managing Childbirth and Fertility in Medieval Europe’, in *Reproduction: Antiquity to the Present Day*, eds. Nick Hopwood, Rebecca Flemming, and Lauren Kassell (Cambridge: Cambridge University Press, 2018), 153–66.

3 Zhongwei Zhao, ‘Deliberate Birth Control under a High-fertility Regime: Reproductive Behaviour in China before 1970’, *Population and Development Review* 23 (1997): 729–67; Cameron Campbell and James Z. Lee, ‘Fertility Control in Historical China Revisited: New Methods for an Old Debate’, *The History of the Family* 15 (2010): 370–85; Cameron Campbell and Satomi Kuruso, ‘Asian Historical Demography’, in *Routledge Handbook of Asian Demography*, eds. Zhongwei Zhao and Adrian C. Hayes (London: Routledge, 2017), 45–63.

4 Nick Hopwood, Rebecca Flemming, and Lauren Kassell, ‘Concluding Reflections’, in *Reproduction*, 666.

5 On Soranus see Ann Ellis Hanson and Monica H. Green, ‘Soranus of Ephesus: *Methodicorum Princeps*’, in *Aufstieg und Niedergang der römischen Welt, 37.2* eds. Wolfgang Haase and Hildegard Temporini (Berlin: De Gruyter, 1994), 968–1075, and Rebecca Flemming, *Medicine and the Making of Roman Women: Gender, Nature, and Authority from Celsus to Galen* (Oxford: Oxford University Press, 2000), 228–46.

6 How much Latin those who entered the imperial elite from the Greek East learnt has been more controversial than the facility of Italian—and other western—aristocrats with Greek: see J.N. Adams, *Bilingualism and the Latin Language* (Cambridge: Cambridge University Press, 2008).

7 On the Hippocratic view see Ann Ellis Hanson, ‘The Medical Writers’ Woman’, in *Before Sexuality: The Construction of Erotic Experience in the Ancient Greek World*, eds. David M. Halperin, John J. Winkler, and Froma I. Zeitlin (London: Routledge, 1990), 309–38; Lesley Dean-Jones, *Women’s Bodies in Classical Greek Science* (Oxford: Oxford University Press, 1994); and Helen King, *Hippocrates’ Woman: Reading the Female Body in Ancient Greece* (London: Routledge, 1998).

8 Soranus (Sor.), *Gynecology* (*Gyn.*) 1.33.

9 Legally a girl could be married at 12 and, despite considerable debate on the topic, it is clear that the daughters of elite families were married off early, even if the global average was more towards the late teens. See Saskia Hin, *The Demography of Roman Italy: Population Dynamics in an Ancient Conquest Society* (Cambridge: Cambridge University Press, 2013), 175–81 for a summary.

10 Sor. *Gyn.* 1.34. All translations are my own. ‘*Sullambanein’* is literally ‘to gasp’ or ‘to hold together’, the action of the womb which underlies the ancient notion of ‘conception’.

11 As collected in Susan Treggiari, *Roman Marriage:* Iusti Coniuges *from the Time of Cicero to the Time of Ulpian* (Oxford: Oxford University Press, 1991), 83–124, though she takes Soranus as descriptive rather than prescriptive.

12 Tacitus, *Annals* 1.41; 12. 2 and 6; 14.1.

13 Sor. *Gyn*. 1.36–41.

14 The substances and forces involved in conception were debated in antiquity, see Rebecca Flemming, ‘Galen’s Generation of Seeds’, in *Reproduction*, eds. Hopwood, Flemming and Kassell, 95–108.

15 See Dean-Jones, *Women’s Bodies*, 170–6.

16 A.J. Wilcox, D. Dunson, and D.D. Baird, ‘The Timing of the “Fertile Window” in the Menstrual Cycle: Day Specific Estimates from a Prospective Study’, *British Medical Journal* 7271 (18 November 2000): 1259–62.

17 Sor. *Gyn* 1.46–7, 48–53 and 54–6 respectively.

18 Ibid. 1.42.

19 Ibid. 1.60.

20 The main manuscript tradition of the Hippocratic *Oath* specifies ‘abortive pessaries’, see Thomas Rutten, ‘Receptions of the Hippocratic Oath in the Renaissance: The Prohibition of Abortion as a. Case Study in Reception’, *Journal of the History of Medicine* 51 (1996): 456–83. On ancient debates about abortion, see Konstantinos Kapparis, *Abortion in the Ancient World* (London: Duckworth, 2002) and Zubin Mistry, *Abortion in the Early Middle Ages, c.500–900* (Woodbridge: Boydell, 2015), 23–55.

21 Sor. *Gyn.* 1.61.

22 Ibid*.* 1.61–62.

23 Ibid*.* 1.63.

24 So, it was hard to draw a line between contraception and abortion in practice: Keith Hopkins, ‘Contraception in the Roman Empire’, *Comparative Studies in Society and History* 8 (1965): 124–51.

25 Sor. *Gyn.* 1.64–5.

26 John M. Riddle, *Contraception and Abortion from the Ancient World to the Renaissance* (Cambridge, MA: Harvard University Press, 1994) and *Eve’s Herbs: A History of Contraception and Abortion in the West* (Cambridge, MA: Harvard University Press 1997).

27 King, *Hippocrates’ Woman*, 132–56, is the most systematic

28 See e.g. Dinesh Kumar, Ajay Kumar, and Om Prakesh, ‘Potential Antifertility Agents from Plants: A Comprehensive Review’, *Journal of Ethnopharmacology* 140 (2012): 1–32.

29 On historically useful notions of efficacy see e.g. Nancy Demand, ‘Did the Greeks Believe in the Efficacy of Hippocratic Treatment—and, if so, Why?’ in eds. Ivan Garofalo et al., *Aspetti della terapia nel Corpus Hippocraticum* (Florence: Olschki, 1999), 139–48, and Laurence M.V. Totelin, *Hippocratic Recipes: Oral and Written Transmission of Pharmacological Knowledge in Fifth- and Fourth-century Greece* (Leiden: Brill, 2009), 219–24.

30 Sor. *Gyn.* 1.60; see also the Hippocratic text *Fleshes* 19 on prostitutes and abortion.

31 Lucretius, *On the Nature of Things/De Rerum Naturae* (*DRN*) 4.1269–1273.

32 Rebecca Flemming, ‘*Quae Corpore Quaestum Facit*: The Sexual Economy of Female Prostitution in the Roman Empire’, *Journal of Roman Studies* 89 (1999): 38–61; Thomas McGinn, *Prostitution, Sexuality and the Law in Ancient Rome* (Oxford: Oxford University Press, 1998).

33 Sor. *Gyn.* 2.10.

34 Ibid. 2.18.

35 See Keith Bradley, ‘Wet-nursing at Rome: A Study in Social Relations’, in ed. Beryl Rawson, *The Family in Ancient Rome: New Perspectives* (London: Routledge, 1986), 201–29; Tim Parkin, ‘The Demography of Infancy and Early Childhood in the Ancient World’, in *The Oxford Handbook of Childhood and Education in the Classical World*, eds. Judith Evans Grubbs, Tim Parkin, and Roslynne Bell (Oxford: Oxford University Press, 2013), 50–7; Claude-Emmanuelle Centlivres Challet, ‘Roman Breastfeeding: Control and Affect’, *Arethusa* 50 (2017): 369–84.

36 Sor. *Gyn*. 4.1–13 (*dustokia*); and see e.g. 3.17 and 40 (inflammation and haemorrhage).

37 Sor. *Gyn* 2.27 (*atonia* of the womb).

38 See Green and Hanson, ‘Soranus of Ephesus’, 970–81 and 1042–61; and Lesley Bolton, ‘Edition, Translation and Commentary on Mustio’s *Gynaecia*’ (PhD thesis, University of Calgary, 2015), 1–21 and 48–102.

39 Chapter 157 in the Greek manuscript ‘index’: Caelius Aurelianus (Cael. Aur.) *Genecia* (*Gen.*) 2.64–65; Muscio 143. I have translated literally, it is unclear whether the formula is pleonastic or if there is some distinction between these terms, see further: Rebecca Flemming, ‘The Invention of Infertility in the Classical Greek World: Medicine, Divinity, and Gender’, *Bulletin of the History of Medicine* 87 (2013): 565–590.

40 Cael. Aur. *Gen.* 2.64.

41 That is the ‘typical use’ rather than ‘perfect use’ figure for the pill provided by the NHS (https://www.nhs.uk/conditions/contraception/how-effective-contraception/). For IVF success rates see the statistics provided by the Human Fertilisation and Embryology Authority: https://www.hfea.gov.uk/treatments/explore-all-treatments/in-vitro-fertilisation-ivf/.

42 UK institutions tend to prefer the 25% ‘unexplained’ figure, e.g. the National Institute for Health and Care Excellence (NICE): https://www.nice.org.uk/guidance/cg156/chapter/Context.

43 See e.g. Aristotle, *Generation of Animals* 723a29, 727b11 and 767a16–25; *History of Animals* 10 636b9–24; Lucr. *DRN* 4.1248–1259; Pliny, *Natural History* 7.57; Galen, *Commentary on Hippocrates’ Aphorisms* 5. 62 (18B 861-8 K).

44 Lucr. *DRN* 4.1251–1256.

45 See e.g. Treggiari, *Roman Marriage*, 435–82; Judith Evans Grubbs, ‘Children and Divorce in Roman Law’, in *Hoping for Continuity: Childhood, Education and Death in Antiquity and the Middle Ages*, eds. Katariina Mustakallio et al. (Rome: Institutum Romanum Finlandiae, 2005), 33–47.

46 Angela Hug, ‘*Fertilitas, Sterilitas* and the Politics of Reproduction at Rome’ (PhD diss., York University, Toronto, 2014), 331–45.

47 See e.g. Artemidorus, *On Dream Interpretation* 1.16, 26, 33 and 35, 2.14, 20, 24 and 37; Ptolemy, *Tetrabiblos* 4.6; *Oracles of Astrampsychus* questions 24, 30, 47 and 59.

48 See e.g. Tamsyn Barton, *Ancient Astrology* (London: Routledge, 1994), 38–63.

49 These are the main Latin and Greek terms, which literally signify a ‘placing out’. The vocabulary is wider, though largely interchangeable.

50 The point is well-made in recent scholarship—William V. Harris, ‘Child-exposure in the Roman Empire’, *Journal of Roman Studies* 84 (1994): 1–22; Mireille Corbier, ‘Child Exposure and Abandonment’, in *Childhood, Class and Kin in the Roman World*, ed. Suzanne Dixon (London: Routledge, 2001), 52–73; Judith Evans Grubbs, ‘Infant Exposure and Infanticide’, in *The Oxford Handbook of Childhood*, eds. Evans Grubbs, Parkin, and Bell, 83–107.

51 Evans Grubbs, ‘Infant Exposure’, 90; see also e.g. Maureen Carroll, *Infancy and Earliest Childhood in the Roman World* (Oxford: Oxford University Press, 2018), 170–6.

52 Jack Caldwell counted infant exposure as a method of ‘fertility control’ without comment: John C. Caldwell, ‘Fertility Control in the Classical World: Was there an Ancient Fertility Transition?’, *Journal of Population Research* 21 (2004): 8. Fabian Drixler, *Mabiki Infanticide and Population Growth in Eastern Japan, 1660–1950* (Berkeley: University of California Press, 2013), 16–9.

53 Unless his father was alive: on the fundamental Roman notion of *patria potestas* see e.g. Richard P. Saller, *Patriarchy, Property and Death in the Roman Family* (Cambridge: Cambridge University Press, 1994); Suzanne Dixon, ‘Family’, in *The Oxford Handbook of Roman Law and Society*, eds. Paul J. du Plessis, Clifford Ando, and Kaius Tuori (Oxford: Oxford University Press, 2016), 461–72.

54 For an outline of Roman inheritance law see e.g. Éva Jakab, ‘Inheritance’, in *Roman Law and Society*, eds. du Plessis, Ando, and Tuori, 498–510.

55 Fr. 15 (Lutz).

56 Longus, *Daphnis and Chloe* 4.24.

57 Ibid. 4.35.

58 *BGU* 1V.1104; Jane Rowlandson, *Women and Society in Greek and Roman Egypt: A Sourcebook* (Cambridge: Cambridge University Press, 1998), no. 129.

59 Justinian, *Digest* 40.4.29 (Scaevola) and see Judith Evans Grubbs, ‘Hidden in Plain Sight: *Expositi* in the Community’, in *Children, Memory, and Family Identity in Roman Culture*, eds. Véronique Dasen and Thomas Späth (Oxford: Oxford University Press, 2010), 294–7.

60 See Beryl Rawson, ‘*Spurii* and the Roman view of illegitimacy’, *Antichthon* 23 (1989): 10–41; Sabine Hübner and David Ratzan (eds.), *Growing Up Fatherless in Antiquity* (Cambridge: Cambridge University Press, 2009).

61 Fostering in the Roman world covered many forms of the raising of others’ children, whether they be foundlings or from elsewhere in the family, enslaved or free: see Hanne Sigismund-Nielsen, ‘*Alumnus:* A Term of Relation that Denotes Quasi-adoption’, *Classica et Mediaevalia* 38 (1987): 142–88.

62 Attempts to identify the so-called *columna lactaria* in Rome as such a place are mistaken: Corbier, ‘Child Exposure’, 62–3; see also Evans Grubbs, ‘Infant Exposure’, 93.

63 Dio, *Discourses* 15.8.

64 Juvenal, *Satires* 6.592–609.

65 Justinian, *Digest* 48.10.19 (Paul) and 1.6.6 (Ulpian).

66 A key point first drawn attention to by J. Ramin and P. Veyne, ‘Droit Romain et société: les hommes libres qui passent pour esclaves et l’esclavage voluntaire’, *Historia* 30 (1981): 472–97; and much discussed since, e.g. Evans Grubbs, ‘Hidden in Plain Sight’, 297–305; Kyle Harper, *Slavery in the Late Roman World, AD 275–425* (Cambridge: Cambridge University Press, 2011), 392–409. Selling your own children into slavery was also prohibited, though selling yourself was possible.

67 Pliny *Letters* 10.65 and 66.

68 Evans Grubbs, ‘Hidden in Plain Sight’, 305–10.

69 Hin, *The Demography of Roman Italy*, 172–209.

70 Adoption (*adoptio*) is the general term, covering both the processes of *adrogatio* (by the power of the people and then emperor) and *adoptio* (by the power of a magistrate): Gaius, *Institutes* 1.98–107; Aulus Gellius 5.19.

71 Jane F. Gardner, *Family and* Familia *in Roman Law and Life* (Oxford: Oxford University Press, 1998), 114–208; Christiane Kunst, *Römische Adoption: Zur Strategie einer Familienorganisation* (Hennef: Clauss, 2005) emphasizes the politics.

72 Women (and children) could be adopted but for different reasons; women could not adopt in this period.

73 Valerius Maximus 77.6. The surrendered son attempted to prevent this by disinheriting his natural father (who retained some rights of inheritance despite the transfer of paternal power) but was overruled by the praetor.

74 Valerius Maximus 5.10.2; see also e.g. Plutarch, *Life of Paulus*; Livy 45.40.6–9. The abundance included daughters, though they joined their husbands’ families.

75 Hug, ‘*Fertilitas*’, 304–23.

76 Sigismund-Nielsen, ‘*Alumnus*’.

77 Made by Anthony Douglas, the chief executive of the UK *Children and Family Court Advisory and Support Service* (Cafcass) in an interview in the *Telegraph* (2 November 2018). Adoption rates continue to fall.

78 See Hanne Sigismund-Nielsen, ‘Slave and Lower-class Roman Children’, in *Childhood and Education in the Classical World*, eds. Evans Grubbs, Parkin, and Bell, 286–300.

79 Zhao, ‘Deliberate Birth Control’, 738–44.

80 Mireille Corbier, ‘Divorce and Adoption as Roman Familial Strategies’, in *Marriage, Divorce, and Children in Ancient Rome*, ed. Beryl Rawson (Oxford: Oxford University Press, 1991), 47–78; Saller, *Patriarchy*.

